# Intimal spindle cell sarcoma masquerading as adult-onset symptomatic pulmonic stenosis: a case report and review of the literature

**DOI:** 10.1186/s13019-017-0654-9

**Published:** 2017-10-30

**Authors:** Arun Manmadhan, Sunil P. Malhotra, Catherine R. Weinberg, Alex Reyentovich, Larry A. Latson, Puneet Bhatla, Muhamed Saric

**Affiliations:** 1 Department of Medicine, Leon H. Charney Division of Cardiology, New York University School of Medicine, New York, NY USA; 2 Department of Cardiothoracic Surgery, Division of Pediatric and Adult Congenital Cardiac Surgery, New York University School of Medicine, New York, NY USA; 3 Department of Radiology, Cardiac and Thoracic Imaging, New York University School of Medicine, New York, NY USA; 4 Department of Pediatrics, New York University School of Medicine, New York, NY USA; 5 Echocardiography Lab, New York University Langone Medical Center, New York, NY 10016 USA

**Keywords:** Intimal sarcoma, Spindle cell sarcoma, Pulmonary artery sarcoma, Pulmonic stenosis, Right ventricular outflow tract obstruction (RVOT), Cardiac tumor, Valvular tumor, Pulmonic valve tumor

## Abstract

**Background:**

Pulmonary artery intimal spindle cell sarcomas are rare and carry with them a poor prognosis and high rate of recurrence. In extremely rare cases, this tumor can infiltrate the pulmonic valve and manifest as adult-onset pulmonic stenosis.

**Case presentation:**

We report an unusual case of a patient with symptomatic, adult-onset severe pulmonic stenosis who was referred for possible balloon valvuloplasty but was subsequently found to have pulmonary artery intimal sarcoma infiltrating the pulmonary valve leading to progressive exertional dyspnea.

**Conclusion:**

The presence of adult-onset pulmonic stenosis should prompt the clinician to investigate further as most cases of pulmonic stenosis are congenital in nature and present early in life. Careful diagnostic evaluation in concert with multimodal imaging should take place to arrive at the correct and challenging diagnosis of sarcoma-induced adult-onset severe pulmonic stenosis. Given the poor prognosis and rapid progression of disease, early diagnosis is crucial.

**Electronic supplementary material:**

The online version of this article (10.1186/s13019-017-0654-9) contains supplementary material, which is available to authorized users.

## Background

In the majority of cases, pulmonic stenosis is congenital in nature and is commonly diagnosed and treated in the pediatric population. The diagnosis of acquired pulmonic stenosis in adulthood is unusual and may represent an oncologic phenomenon and warrants a thorough workup to identify the etiology of the stenosis. Here we present a case of a patient with adult-onset pulmonic stenosis due to pulmonary artery intimal sarcoma and the multimodal and multidisciplinary efforts needed to diagnose and treat the patient.

## Case presentation

A 79-year-old man with a 1-month history of progressive dyspnea and exercise intolerance was found to have severe pulmonic valve stenosis and was referred to our institution for balloon valvuloplasty. Prior medical history included coronary artery disease, hypertension and chronic kidney disease.

**Fig. 1:**
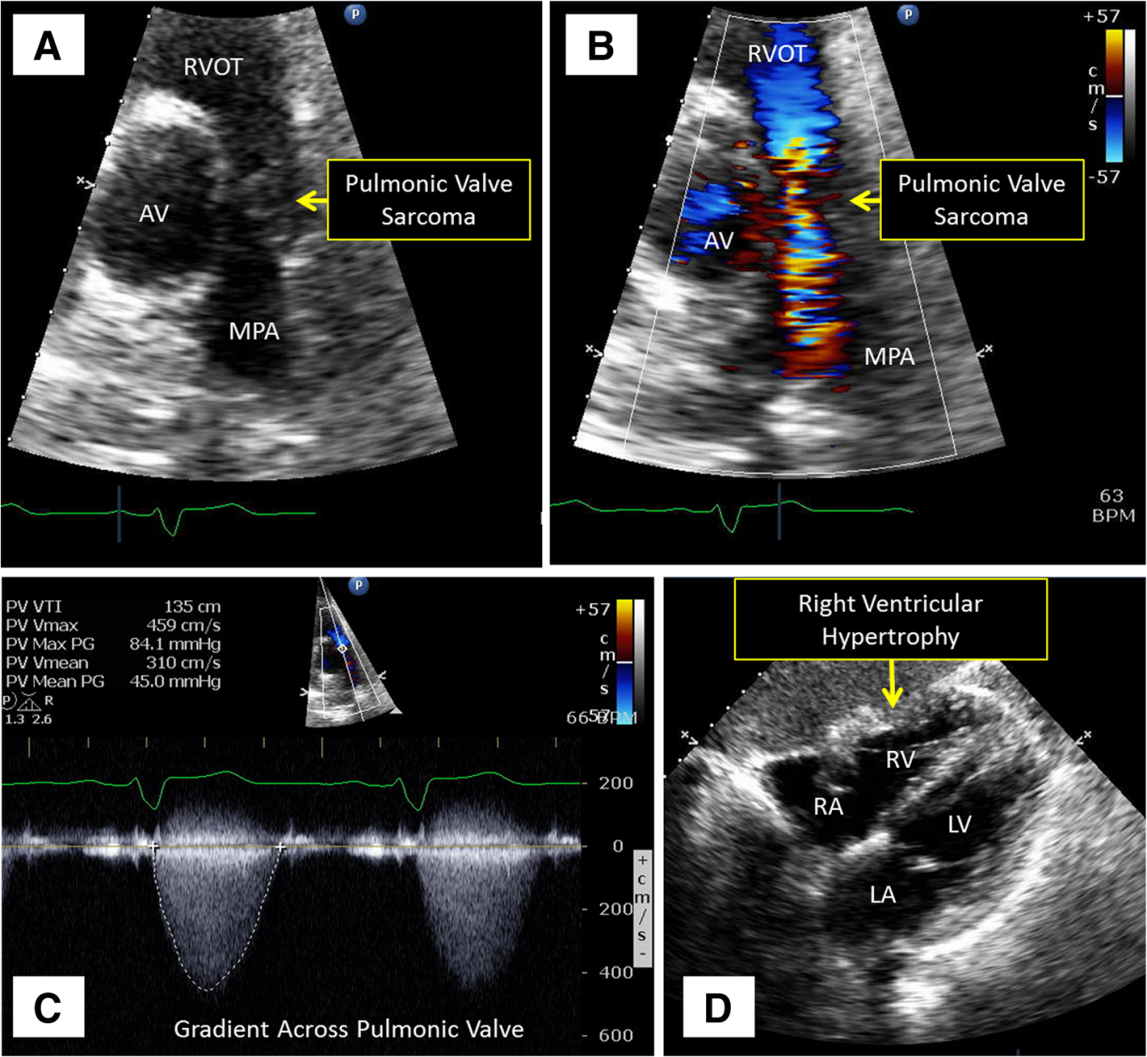
Transthoracic Echocardiogram. 2D TTE image demonstrates the sarcoma infiltrating the pulmonic valve (Panel **a**) leading to marked flow acceleration across the pulmonic valve on color Doppler imaging (Panel **b**) and spectral Doppler tracing with a peak velocity of 4.6 m/s and a peak gradient of 84 mmHg (Panel **c**). The presence of marked right ventricular hypertrophy (Panel **d**) implies chronic pressure overload of the right ventricle. Abbreviations: AV, aortic valve; LA, left atrium; LV, left ventricle; MPA, main pulmonary artery; RA, right atrium; RV, right ventricle; RVOT, right ventricular outflow tract

At the referring institution, the physical exam was notable for a III/VI systolic murmur best appreciated in the left upper sternal border. An electrocardiogram (EKG) demonstrated sinus rhythm with a right bundle branch block and a chest x-ray was normal. Transthoracic echocardiogram (TTE) was reported as demonstrating severe pulmonic stenosis with a peak gradient of 100 mmHg and peak velocity of 5 m/s associated with right ventricular hypertrophy, severe tricuspid regurgitation and severe right atrial dilatation (Fig. 1, Additional files 1, 2, 3

**Additional file 1: Movie S1.** Movie of Fig. 1, Panel A demonstrating sarcoma infiltrating the pulmonic valve. (MP4 1935 kb)

**Additional file 2: Movie S2.** Movie of Fig. 1, Panel B demonstrating flow acceleration across the pulmonic valve. (MP4 2304 kb)

**Additional file 3: Movie S3.** Movie of Fig. 1, Panel D in short-axis view showing marked right ventricular hypertrophy. (MP4 3092 kb)).

**Fig. 2:**
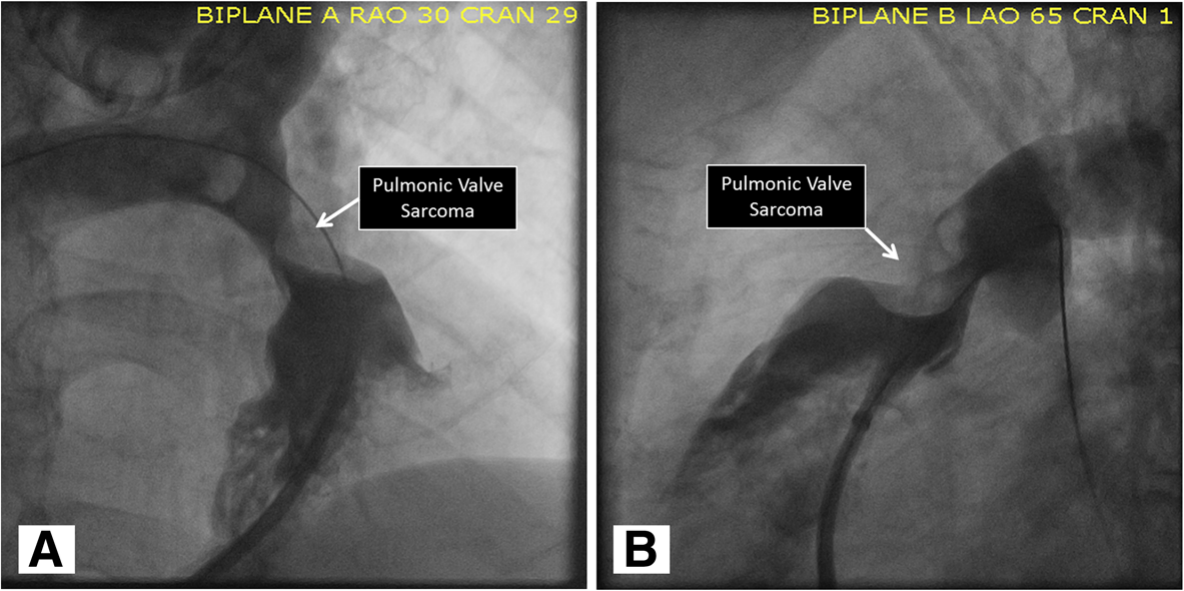
Pulmonary Angiogram. Biplane pulmonary angiogram in the right anterior oblique (Panel **a**) and left anterior oblique (Panel **b**) view demonstrates flow obstruction at the level of the pulmonic valve consistent with the subsequent diagnosis of spindle cell sarcoma

The patient was taken to the catheterization lab for planned balloon valvuloplasty but the procedure was canceled after angiography revealed an obstructive mass in the right ventricular outflow tract (RVOT) of unclear etiology extending into the main pulmonary artery (Fig. 2, Additional files 4, and 5

**Additional file 4: Movie S4.**
Movies of Fig. 2, Biplane angiogram in right anterior oblique. (MP4 2764 kb)

**Additional file 5: Movie S5.**
Left anterior oblique view (movie S5) revealing flow obstruction at the level of the pulmonic valve. (MP4 3061 kb)). Contrast-enhanced computed tomographic (CT) angiogram and gadolinium-enhanced cardiac magnetic resonance imaging (MRI) were obtained to better characterize the obstructive mass.

**Fig. 3:**
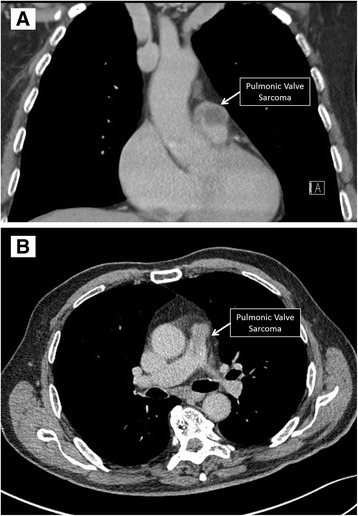
CT. Contrast-enhanced chest CT demonstrates spindle-cell sarcoma at the level of the pulmonic valve on a coronal (Panel **a**) and axial cut (Panel **b**)

CT angiogram showed a fixed intravascular defect within the proximal main pulmonary artery arising from the pulmonic valve with mixed enhancement properties suggesting a thrombus versus a vascular mass; no other filling defects were noted (Fig. 3).

**Fig. 4:**
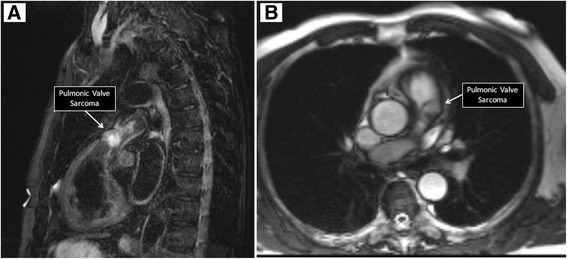
MRI. T2 weighted sagittal image showing spindle-cell sarcoma (Panel **a**). Still image from a TruFisp cine sequence, showing the location of the mass in the pulmonary artery (Panel **b**)

Cardiac MRI revealed a T1 and T2 hyperintense ovoid mass measuring 2.0 × 1.5 cm, and centered at the lateral aspect of the pulmonary valve in the region of the anterior sinus of Valsalva. The mass extended into the pulmonary artery resulting in severe pulmonic stenosis (Fig. 4). Neither CT nor MRI demonstrated malignant lesions anywhere else in the body.

**Fig. 5:**
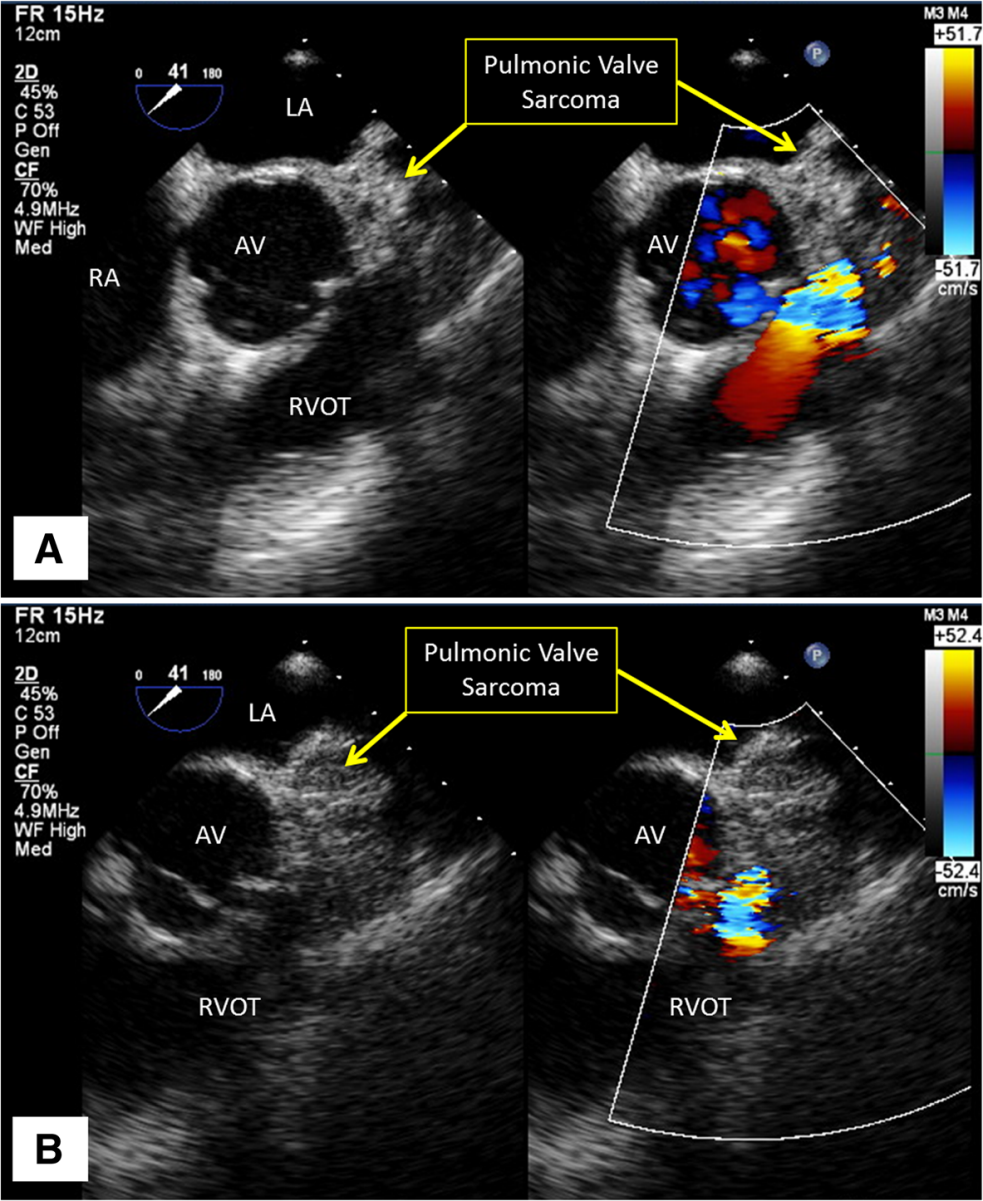
Intraoperative TEE. Intraoperative midesophageal TEE view at two short-axis levels of the aortic valve demonstrates a large mass obstructing the pulmonic valve which was subsequently shown to be spindle cell sarcoma. Abbreviations: AV, aortic valve; LA, left atrium; LV, left ventricle; MPA, main pulmonary artery; RA, right atrium; RVOT, right ventricular outflow tract

Given the CT and MRI findings, the mass was presumed to be a primary cardiac tumor and decision was made to proceed with surgical resection. A preoperative transesophageal echocardiogram (TEE) corroborated CT and MRI findings (Fig. 5, Additional files 6, and 7

**Additional file 6: Movie S6.** Movie of Fig. 5, Panel A showing a large mass obstructing the pulmonic valve. (MP4 4720 kb)

**Additional file 7: Movie S7.** Movie of Fig. 5, Panel B showing a large mass obstructing the pulmonic valve. (MP4 4218 kb)).

**Fig. 6:**
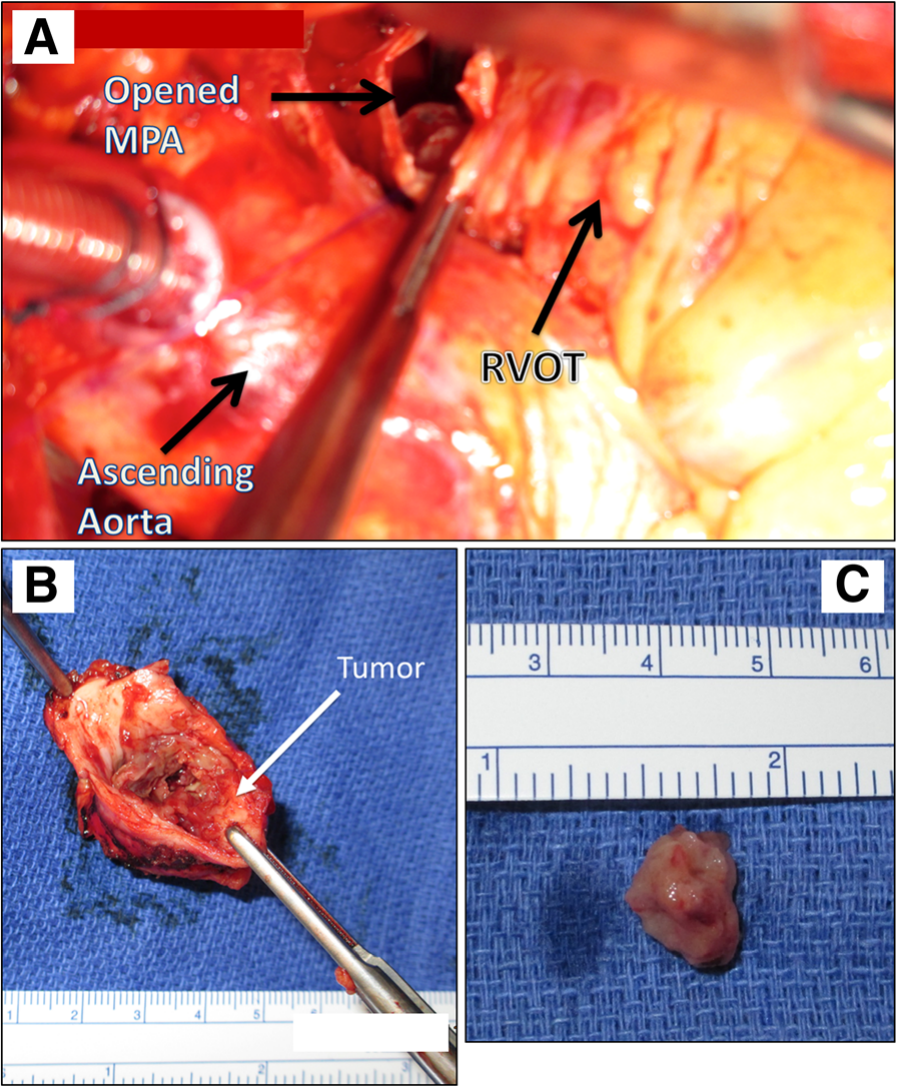
Surgical Specimen. After the main pulmonary artery (MPA) was opened, the tumor was visualized (Panel **a**). En block resection of the MPA, pulmonary valve and distal RVOT muscle (Panel **b**) demonstrates tumor involvement. Resected tumor is seen in (Panel **c**)

**Fig. 7:**
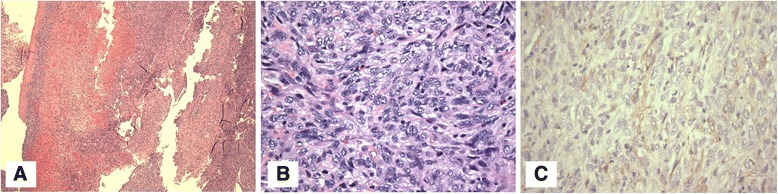
Histopathology. Panel **a** Haematoxylin and Eosin (H&E) stained sections show spindled tumor cells lining the intimal surfaces of the pulmonary artery and pulmonic valve as well as forming nodules with infiltrative borders that are arranged in fascicles (original magnification 40×). Panel **b** Magnified H&E section demonstrates a high degree of nuclear atypia (original magnification 400×). Panel **c** Immunohistochemical staining demonstrates tumor cells that are positive for smooth muscle actin (SMA) (original magnification 400×)

The patient underwent median sternotomy and longitudinal arteriotomy in the main pulmonary artery (MPA) that showed a fungating mass involving the pulmonary annulus and MPA with extension into the RVOT (Fig. 6). The tumor was resected en bloc and the RVOT was reconstructed using a pulmonary homograft. Surgical histopathology revealed increased mitotic activity and nuclear atypia. Immunohistochemical staining was positive for smooth muscle actin (SMA) consistent with intimal spindle cell sarcoma (Fig. 7).

Postoperatively, a TTE demonstrated normal pulmonary valve function and his dyspnea resolved. He was then referred for chemotherapy with pazopanib, a tyrosine kinase inhibitor, but was lost to follow up.

## Discussion

The vast majority of cases of pulmonic stenosis in children and adults are due to congenital heart disease. Acquired pulmonic stenosis is rare with carcinoid syndrome, rheumatic fever and infective endocarditis accounting for the majority of cases [1]. Very rarely, pulmonic stenosis and RVOT obstruction are caused by tumors.

The majority of cardiac tumors are malignant and metastases of extracardiac malignancies [2]. The incidence of primary cardiac tumors, whether benign or malignant, is very low (approximately 0.02% in the general population) [2]. Most primary cardiac tumors are benign; malignant tumors account for only 15% of all primary cardiac tumors. Sarcomas are by far the most common primary cardiac malignancies [3, 4].

Here, we report a case of a malignant intimal sarcoma infiltrating the pulmonic valve, a very rare cause of adult-onset severe pulmonary stenosis and otherwise unexplained progressive exertional dyspnea. The patient in question was initially referred to our institution for balloon valvuloplasty – the treatment modality of choice for symptomatic, congenitally-acquired pulmonic stenosis. However, it is highly atypical for congenitally-acquired pulmonic stenosis to present late in life with symptoms. Therefore, the presentation of adult-onset symptomatic pulmonic stenosis should prompt a nuanced diagnostic workup to identify the correct diagnosis and optimal choice of treatment.

Intimal sarcoma was first described in 1923 by Moritz Mandelstamm [5]. Such tumors are thought to arise from multipotent mesenchymal cells of the intima of the great arteries [6]. Between 1923 and 2012, less than 200 cases of intimal sarcoma involving the great arteries have been described with the majority of reports focusing on histological features and surgical management [7]. When intimal sarcomas arise from the main pulmonary artery they tend to extend into the pulmonary artery branches and rarely involve the pulmonary valve and RVOT [8–13].

It has previously been reported that intimal sarcomas have a female predominance. However, a more recent study has shown an equal sex distribution with an average age of disease onset of 49.3 years (range 13-81 years) [14]. By the time of presentation, the disease is usually advanced and carries a poor prognosis with a median survival of only 17 months [15].

Establishing the diagnosis of intimal sarcoma is challenging. Pulmonary artery intimal sarcomas often present with cough, dyspnea and chest pain associated with radiological features suggestive of a thrombus or obstructive mass in the pulmonary artery [7]. The most common finding on physical examination is a systolic ejection murmur which is seen in 44% of patients [14]. The overall clinical presentation of pulmonary artery intimal sarcomas mimic pulmonary embolism and there are several reports in the literature of intimal sarcoma masquerading as pulmonary embolism [16–19].

The current diagnostic gold-standard is tissue sampling and immunohistochemical analysis. Intimal sarcomas can radiographically mimic thrombus and drastically alter clinical decision making. Therefore, preoperative, multimodality imaging is of paramount importance. Doppler echocardiography is the screening tool of choice and should be considered in all patients with suspected intracardiac masses as it allows for identification and characterization of the mass based on potential hemodynamic consequences [20]. Transthoracic and transesophageal echocardiography have 93% and 97% sensitivity in detecting primary cardiac tumors, respectively [21]. Intimal sarcomas are highly vascularized tumors that readily demonstrate hyperenhancement with contrast perfusion echocardiography [22]. On the other hand, stromal tumors and thrombi demonstrate hypoenhancement. Other TTE findings suggestive of intimal sarcoma include a right sided location, right ventricular hypertrophy and a bulging or ovoid mass with involvement of the pulmonic valve or RVOT causing pulmonic stenosis [23].

Other imaging modalities that are useful in diagnosing intimal sarcoma include CT, cardiac MRI and positron emission tomography (PET) scans. On CT, intimal sarcomas are more likely to have a heterogeneous appearance with smooth, contiguous areas of spread, often expanding beyond the vessel wall, in contrast to thrombus [19]. On MRI, intimal sarcomas are more likely to demonstrate enhancement and vascularity after administration of contrast that allows for accurate anatomic localization. Finally, PET scans can aid in diagnosis and prognosis by showing tumor size, spread and distribution.

Radical surgical resection remains the only definitive mode of treatment. Without surgery the mean survival rate is 1.5 months and with surgery the chance of recurrence and spread remains high [15]. In certain cases, cardiac transplantation may prolong survival compared to treatment with local resection [24]. There are some reports of improved survival with adjuvant chemotherapy following resection, however, data are limited [25–27].

## Conclusion

In summary, in patients presenting with symptomatic, adult-onset pulmonic stenosis with radiographic features of an obstruction in the pulmonary artery or RVOT, intimal sarcoma should be a consideration on the differential diagnosis. Early diagnosis is crucial as this disease is rapidly progressive and has a poor prognosis.

## Abbreviations

CT: Contrast-enhanced computed tomographic
EKG: Electrocardiogram
MPA: Main pulmonary artery
MRI: Magnetic resonance imaging
PET: Positron emissions tomography
RVOT: Right Ventricular Outflow Tract
SMA: Smooth muscle actin
TEE: Transesophageal echocardiogram
TTE: Transthoracic echocardiogram
